# Simple analysis of gel images with IOCBIO Gel

**DOI:** 10.1186/s12915-023-01734-8

**Published:** 2023-10-20

**Authors:** Jaak Kütt, Georg Margus, Lauri Kask, Triinu Rätsepso, Kärol Soodla, Romain Bernasconi, Rikke Birkedal, Priit Järv, Martin Laasmaa, Marko Vendelin

**Affiliations:** 1 Laboratory of Systems Biology, Department of Cybernetics, Tallinn University of Technology, Tallinn, Estonia; 2 Applied Artificial Intelligence Group, Department of Software Science, Tallinn University of Technology, Akadeemia 21, Tallinn, 12618 Estonia

**Keywords:** Data analysis, Reproducibility, FAIR, Western blotting, Southern blotting, Isoelectric focusing

## Abstract

**Background:**

Current solutions for the analysis of Western Blot images lack either transparency and reproducibility or can be tedious to use if one has to ensure the reproducibility of the analysis.

**Results:**

Here, we present an open-source gel image analysis program, IOCBIO Gel. It is designed to simplify image analysis and link the analysis results with the metadata describing the measurements. The software runs on all major desktop operating systems. It allows one to use it in either a single-researcher environment with local storage of the data or in a multiple-researcher environment using a central database to facilitate data sharing within the research team and beyond. By recording the original image and all operations performed on it, such as image cropping, subtraction of background, sample lane selection, and integration boundaries, the software ensures the reproducibility of the analysis and simplifies making corrections at any stage of the research. The analysis results are available either through direct access to the database used to store it or through the export of the relevant data.

**Conclusions:**

The software is not only limited to Western Blot image analysis and can be used to analyze images obtained as a part of many other widely used biochemical techniques such as isoelectric focusing. By recording the original data and all the analysis steps, the program improves reproducibility in the analysis and contributes to the implementation of FAIR principles in the related fields.

**Supplementary Information:**

The online version contains supplementary material available at 10.1186/s12915-023-01734-8.

## Background

When faced with the task of analyzing Western Blot [1] membrane images, in our experience, researchers usually use either proprietary software solutions built by imaging hardware manufacturers or the open-source ImageJ Gels plugin [2]. Unfortunately, these choices have significant shortcomings. As argued below, the major shortcoming of both software choices is related to the reproducibility of the analysis.

Proprietary software can be a convenient solution. However, such software is frequently linked with specific hardware and the data analysis can usually be reproduced only using the same software package. This limits the ability to reproduce the analysis to the users having the same hardware platform for imaging. If, in the proprietary software, advanced algorithms have been used for background subtraction or data processing, those algorithms are usually not described in a way that other researchers could take the same images and obtain the same results. In addition, the workflow with the image processing and data selection steps is usually not available in a documented format that can be used by other tools except the original software. As a result, the proprietary software for gel analysis often seems like a “black box,” and the users have to contend with the hope that the analyses are done correctly [3, 4].

As an alternative to proprietary software packages, researchers can use the free, open-source ImageJ Gels plugin [2]. The open-source solution means that, in principle, researchers have full control over the image processing and analysis steps, allowing one to fully document the analysis and make it reproducible by others. However, in practice, it is very labor-intensive to document all the analysis steps properly. Namely, one has to fully describe the overall image background subtraction, lanes selection, and integration of image intensities corresponding to each lane with the corresponding baseline selection. As a result, to our knowledge, anyone rarely does this documentation, and this leads to a reduction of the analysis reproducibility. An additional obstacle occurred, when some steps in the analysis had to be redone. For example, if the lane selection had to be adjusted, the user would have to repeat all the analysis steps and find and update the data in a spreadsheet or database before repeating all the statistical analyses. In our experience, this approach was time-consuming and prone to human errors.

A similar problem is encountered in many other popular biochemical techniques that lead to a relation between the sample and its signal, as in Western Blot. This is the case for all gel electrophoresis experiments, where each sample is allocated a well, and the applied electrical field pulls the sample through the gel, forming a lane. Within each lane, molecules within the sample are separated by charge and size. This technique is used in, for example, Western blotting (protein separation by size), Southern blotting (DNA separation by size), Northern blotting (RNA separation by size), and isoelectric focusing (separation by isoelectric point). Considering the popularity of these techniques, the inability to analyze the data in a reproducible manner contributes to the overall reproducibility crisis in science [5].

To address the shortcomings of the current solution, we designed a new open-source software for image analysis applicable for Western blot and similar techniques. During the design, the following aims were addressed. The software should be easy to use on the leading PC platforms. The software should facilitate the implementation of FAIR (findability, accessibility, interoperability, and reusability) principles [6]. In particular, the software should record all analysis steps by keeping the user selections in a form to retrace and correct them. In addition, the software should be able to store data centrally to facilitate efficient collaboration between researchers, link obtained data to the samples, and export the results for sharing.

In line with the popular ImageJ Gels plugin naming, our software was named IOCBIO Gel and the image analysis as well as sample description is using the term *gel* to describe the physical object that is analyzed. Note that this is not always correct as in the case of Western Blot, where images are obtained from a membrane after the transfer from a gel. However, for simplicity, the term *gel* was used in the user interface and the general description below regardless of the specific experimental protocol and corresponding sample.

## Implementation

The developed software, IOCBIO Gel, is written in Python and uses Qt for the graphical user interface. This choice was based on the following. Qt has support for all the major desktop platforms allowing us to target researchers irrespective of their choice of operating system. Python was selected as it is one of the most popular programming languages, which simplifies maintenance and reviewing of the software, reduces the learning curve for new contributors, and allows to benefit from the large selection of scientific libraries in Python. In particular, we used scikit-image [7] for image processing and PyQtGraph for plotting.

When selecting a solution to access original and analyzed data, we targeted the scenarios where the researcher uses the software either as a stand-alone solution on a PC or shares the data within the working group. For original data and gel images, we provided support for loading them either from a selected folder on the PC or from the central image database provided by OMERO [8]. Through the selected folder, we targeted users that keep their images locally or shared via local network file systems. While OMERO is designed for the central storage of microscopy data, we found it very useful for gel images allowing users to access those centrally. For analysis results and keeping the records regarding all analysis steps, we connect to a database through the SQLAlchemy library [9]. This library provides uniform access to local and central databases. As a result, we can keep analysis data locally using SQLite or centrally in PostgreSQL. To work with the analysis results, users can either export the results into a spreadsheet or directly fetch the data from the database.

The database layout has been designed to simplify the usage of the software in an environment where a central database is used to keep experimental results from different types of experiments, such as when a central laboratory database is shared by multiple primary analysis tools [10, 11] and management systems [12–15]. For that, we isolated the data stored by the software using PostgreSQL schema—named collection of tables and views. To provide the link between sample analysis results, the software generates a table allowing users to enter the sample ID for each gel lane. This table can be further constrained by the user using applicable database software to link the entered sample ID with the table describing the sample.

The software was developed in an open manner through a publicly accessible software repository allowing the users to give direct feedback to the developers regarding the encountered issues and feature requests. The application is released under an open-source license, GPLv3.

## Results and discussion

### User interface

When designing the user interface, we had several aims. First, we aimed to reflect the connections between the physical sample, its location in the gel lane, gel images, and analysis results. That way, the users obtain not just the intensities of the peaks but also the data that links their sample to the protein content of interest or some other similar readout. The expectation is that the obtained data can be immediately analyzed using statistical programs. Second, we prioritized streamlining the operations that are frequent by making them accessible first.

**Fig. 1:**
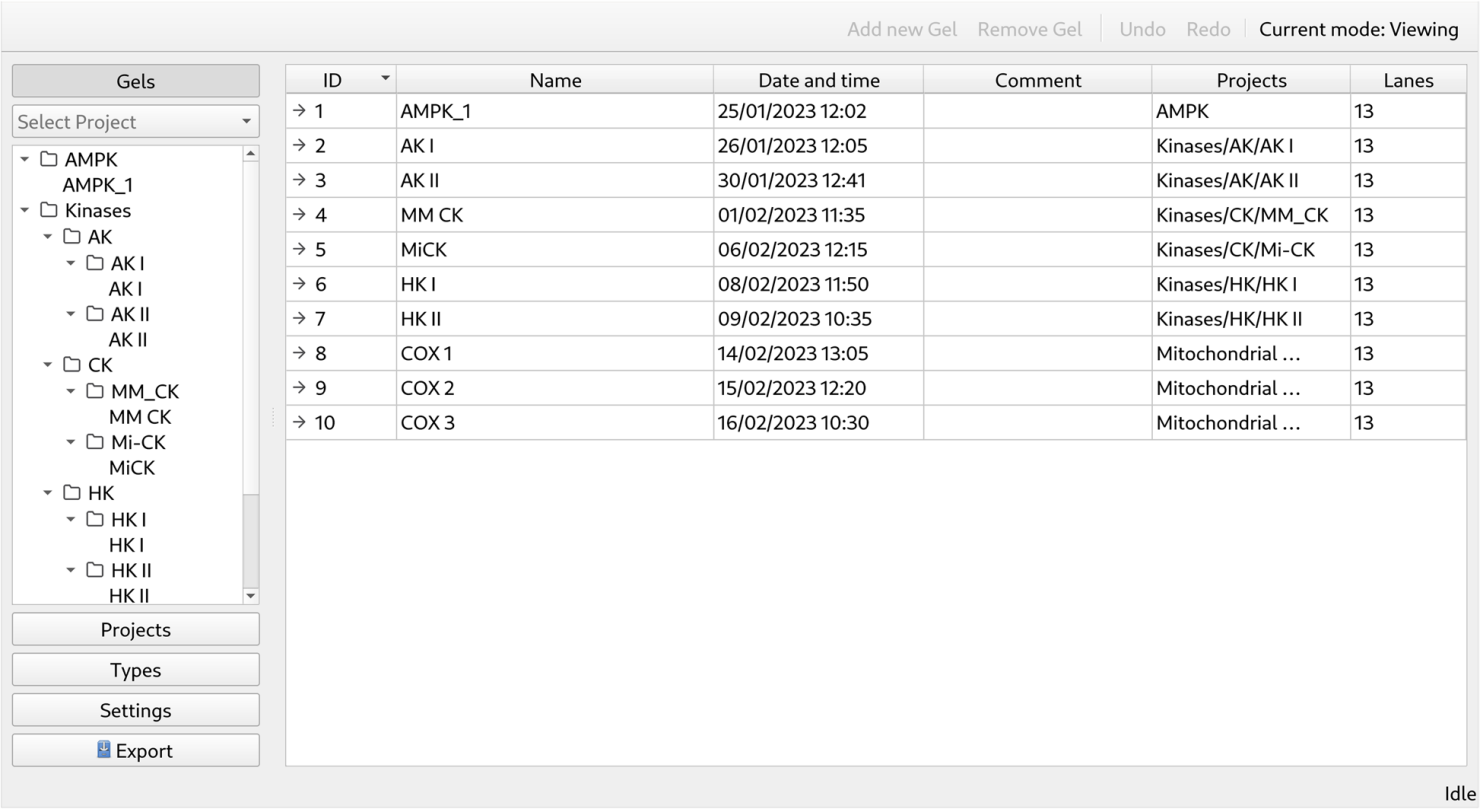
User interface of IOCBIO Gel. The application runs with a single window, which is partitioned to the main action area, application action type selection on the left (selector), the toolbar (top), and the status bar (bottom). The screenshot shows the default view after the start of the application

The overall user interface (Fig. 1) consists of a single window, which is partitioned into the main action area, the application action type selection on the left (*selector*), accompanied by the toolbar (top) and status bar (bottom). To facilitate sharing the data within a research group and to avoid accidental analysis alteration, the application runs in *Viewing* mode by default. In this mode, the user can browse through projects and look at the images and their analysis steps without changing any data. To alter the analysis or enter new data, the user has to switch to *Editing* mode.

### Workflow

**Fig. 2:**
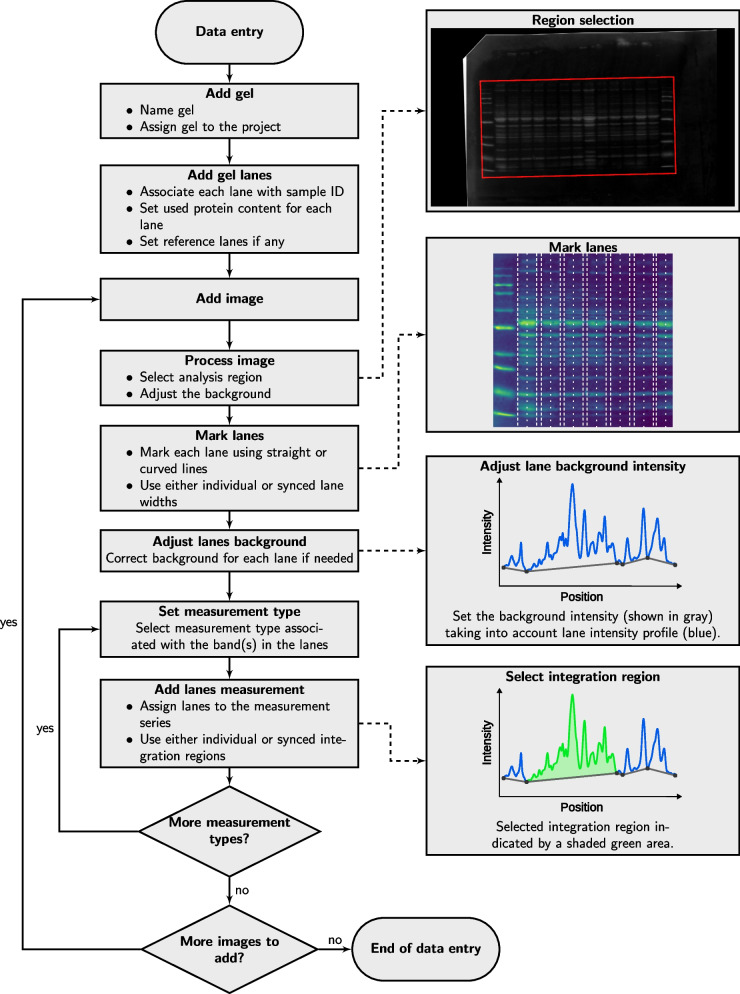
Gel image analysis workflow shown for new gel data entry. Steps include metadata entry at the beginning of the workflow, addition of images, processing them, assigning regions to lanes and performing measurements. Insets, linked with the main workflow scheme with the dashed arrows, illustrate different phases of the data entry

The overall workflow scheme is shown in Fig. 2 and includes entering metadata in addition to the analysis of the images, as described below.

As one of the first steps in analyzing the experiments, the user has to define the context. First, the user defines the types of measurements performed on a physical gel. For example, this could be the detection of a protein of interest using a specific antibody, overall protein content determination using staining, etc. Next, experiments on gels are usually a part of a larger project. Here, the user can handle project information in two ways. When using the same database for multiple projects, for example, in a central laboratory database or keeping all user’s experiments together, we recommend defining projects in the software. Similar to file systems, the projects can form a hierarchy allowing users to partition the measurements within one project into sub-projects. In the central database, we found it advantageous to position projects under each user’s meta-project, making it easier for users to analyze and review their work. As an alternative to the definition of a project, the user can also use separate databases, with each database covering one project only. However, having separate databases would require users to redefine measurement types in each database again, making it inconvenient to switch between the projects.

With the context of the experiment defined, users can start entering the data related to a gel. Users can link a gel to one or multiple projects, as they see fit. Each physical gel is a container of samples that are allocated in gel lanes. As the next step in the analysis, the user is expected to describe each lane and relate it to the samples through their sample ID and the amount of protein in the sample. Some samples on a gel can be assigned as reference samples. This is used to compare samples between different gels, in which case the user relates measurements from the samples to a reference sample present in all these gels.

After the gel is described, the user can insert the images of that gel. In Western blotting, we usually take multiple images of the same membrane. Some are done on the full membrane, such as Ponceau staining, and some on the parts of a membrane, such as after cutting it into smaller subsections for labeling with different antibodies. All the images from the same membrane should be defined together. With a gel image added, it is analyzed by opening it in the application. Image analysis is done by selecting a gel on the image, subtracting the background, manually positioning the gel lanes on the image, and adjusting the baseline for each lane. With the lanes identified on the image and the baseline set, the user must link a measurement type to the image. With the measurement type linked or added to the image, the user can select a section of the intensity curve that is integrated to determine analysis results. Each image could have multiple measurements allowing users to analyze different intensity peaks separately. In addition, each measurement can be marked either as successful or not. Image analysis steps are demonstrated in Fig. 2.

While image analysis is performed manually, we have found that it does not take much time. The application supports different background subtraction algorithms, including rolling ball, that is rather effective for such cases as Ponceau staining. Lanes can be straight or curved, with the same width throughout the gel or individually adjusted. All these options allow users to find the best way to analyze their data in a way that fits their specific experiment. As all steps are recorded, it is easy to come back at any stage of the analysis and adjust it, for example, change the width of the lane, intensity baseline, or section on the intensity curve that is integrated.

### Data analysis

Users can obtain the analysis results in either *raw* or *normalized* form. Here, the *raw* measurement data would contain an integrated intensity corresponding to a selected area of the measurement. This integrated intensity is not normalized. To take into account the amount of loaded sample in a lane, the user would have to divide the intensity by the protein content as a part of the further statistical analysis. As an alternative, *normalized* measurement values are available. Those values are obtained from the raw measurement values by relating these measurements to the measurements on the reference lanes. For that, the average reference measurement value is found by calculating the average for all reference samples on the image *r*, taking into account their protein content:

1 $$r=\frac{1}{R}\sum\limits_{i=1}^{R}\frac{{v}_{i}}{{m}_{i}},$$

where *R* is the number of successful reference samples in the image (could be just one), *v* and *m* correspond to the measurement value and protein content of the lane, respectively, and *i* corresponds to an index of the reference lanes that are marked as successful measurements. As soon as the reference value *r* is known (Eq. 1), the *normalized* measurement value *w* for each lane *j* would be

2 $${w}_{j}=\frac{1}{r}\frac{{v}_{j}}{{m}_{j}}.$$

To get the measurement values v for each lane, the application first applies image cropping and background subtraction. All further analysis is performed on the background-subtracted image. Each lane is marked as a two-dimensional object on the image. As protein separation is expected to be according to the molecular weight, the vertical direction of the image, we first integrate the image intensity of the lane along its width. The result of this integration is shown to the user as a lane intensity profile graph which is intensity as a function of the image’s vertical coordinate (Fig. 2 inset with lane intensity profile). This approach allows users to select curved lanes and preserves lane region selection if the lane has been moved. As a limitation, it does not allow to select lanes that would be horizontal at any of its segments. Next, the user can adjust the background reference for each lane separately. This is needed when the image background subtraction cannot fully take into account the heterogeneity of the background. At the last stage, after the user has selected integration bounds in the vertical direction, the measurement value for the lane is found by integration of the difference between the lane intensity and background.

### Database structure and data export

The database schema layout is designed to ensure data consistency and flexibility in relations between gels and projects. For users, the data access was simplified through three views: a list of projects with their full path, a list of reference measurements for all gels, and a list of normalized values for all measurements. By using relationships between the tables and these views, it is easy to fetch the data from the database directly using SQL to limit the fetched records. Example SQL statements for fetching the data are given in the software repository and are referred to in the user manual. As some statistical analysis programs or data analysis frameworks, such as R, allow to import data from the databases directly, it is possible to use the provided SQL statements in the statistical analysis.

As an alternative to fetching the data from the database, users can export all data or data from a specific project to a spreadsheet. The spreadsheet will contain the same information as in the database and will be structured to simplify data analysis using statistical software. Depending on whether relative or absolute values are of interest, users can analyze raw data or normalized measurements. Note that the raw data does not have normalization to the protein content and has to be normalized by users. This is in contrast to the normalized measurements sheet, where protein content was taken into account.

### Validation

**Fig. 3:**
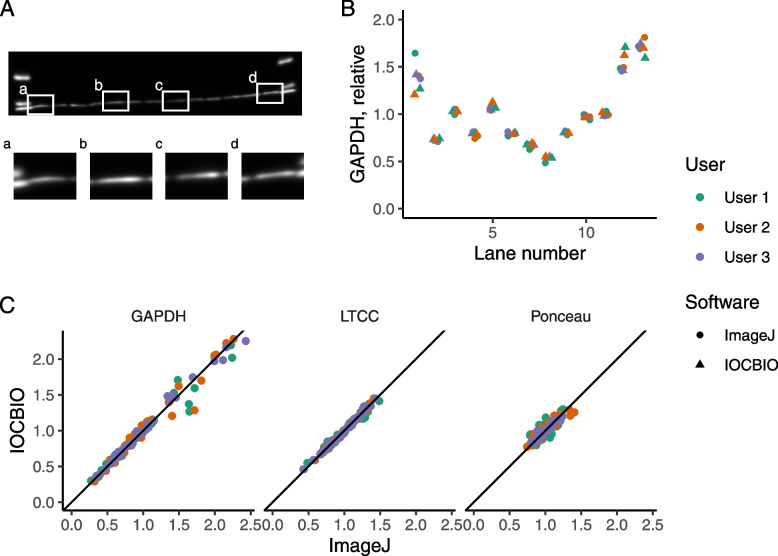
Comparison of analysis by IOCBIO Gel and ImageJ Gel plugin. **A** A representative example of a Western blot membrane labeled with primary and secondary antibodies to assess GAPDH expression (image acquired as a part of [16]). Insets a–d illustrate bands that are either easy to analyze due to clear separation between the lanes (b, c) and that are more complicated due to overlap with the ladder on the left (a) or on the right (d). **B** Results of the expression estimation performed by three users using either IOCBIO Gel or ImageJ Gels plugin to analyze the image shown in A. Notice the very small spread of the estimations in the most of the lanes and the larger spread on the first (shown in inset *a* of **A**) and the two last lanes (inset *d* in **A** corresponds to the last lane). **C** Expression of GAPDH, L-type calcium channel (LTCC), and estimation of protein loading using the Ponceau staining performed by IOCBIO Gel and ImageJ Gels plugin done on three separate membranes and by three different users (analyzed images acquired as a part of [16]). For comparison, the diagonal line corresponding to the equality in estimation is shown in black. Each dot on the plots corresponds to two estimations performed by specific user (shown by color) for the same lane on a gel using IOCBIO Gel (*y*-axis) and ImageJ Gels plugin (*x*-axis). Notice that the dots are distributed along the diagonal line with only small, occasional deviations. This demonstrates that the estimations by both software solutions are the same

To test the software, we compared the estimation of expression levels and protein content performed by IOCBIO Gel and ImageJ Gels plugin, an established solution in the field. For testing, we used images acquired as a part of our earlier study [16] and asked three users to analyze the same images using both software solutions (Fig. 3). The used images are available as an additional file (see Additional file 1). Users obtained results that were similar to each other regardless of the software used. As illustrated in the example on Fig. 3A and B, estimates were mainly influenced by the complexity of determining the band border. For cases, where the bands were relatively well separated, as in the middle section of gel membrane in Fig. 3A, the spread of estimations was very small (Fig. 3B). In contrast, for bands close to the ladder with the signal of the ladder interfering with the band, the estimates had a larger deviation (Fig. 3B).

According to our tests, IOCBIO Gel and ImageJ Gels plugin resulted in very close estimates of the expression or protein content. As shown in Fig. 3C, when plotted against each other, estimates by the different software solutions were located next to the equality line. Pearson’s product-moment correlation between the estimates was statistically significant for all types of measurements (*p* < 0.001) with *r* values of 0.99 (95% CI [0.98, 0.99]) for GAPDH, 0.99 (95% CI [0.99, 1.0]) for LTCC, and 0.89 (95% CI [0.85, 0.92]) for the Ponceau staining. The smaller correlation *r* for Ponceau staining was caused by curved lanes on the gel membranes and usage of larger regions when analyzing by IOCBIO Gel than in ImageJ Gels plugin, as done by Users 1 and 2. Such analysis of larger lane region was possible in IOCBIO Gel as it supports curved lanes. When done using the same regions of the images by the both software solutions, as done by User 3, the correlation *r* for Ponceau staining was 0.96 (95% CI [0.92, 0.98]).

In sum, as shown by the analysis, the estimation of the expression and protein content was the same in IOCBIO Gel and ImageJ Gels plugin.

### Reproducibility

In terms of reproducibility, there are two aspects that we addressed.

First, as IOCBIO Gel keeps all analysis steps in its database automatically, the analysis is reproducible in the sense that one can always open the old analysis and obtain the same result. This is in contrast to ImageJ Gels plugin where, as already explained in introduction, to our knowledge, users rarely record all the steps in such a detail that others could reproduce exactly the same results. So, as a result, there will be always some variability induced by the lack of analysis steps information.

**Fig. 4:**
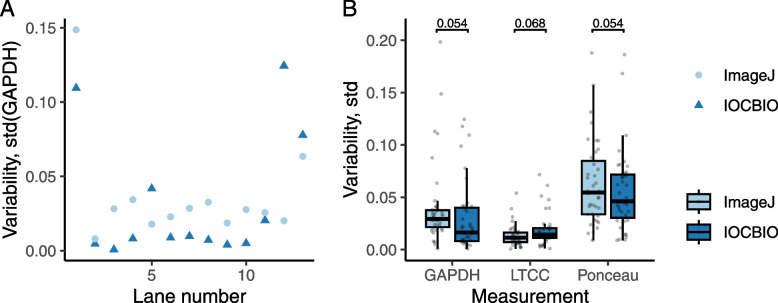
Variability of the analysis by IOCBIO Gel and ImageJ Gel plugin. Variability was estimated as the standard deviation between analysis results performed by different users. **A** Using the image from Fig. 3A, the variability was found for each gel lane when users were either using IOCBIO Gel or ImageJ Gels plugin. **B** Summary of variability estimates for all the gel membrane images that were used to estimate either GAPDH, LTCC, or protein content via Ponceau staining. The variability was found to be not statistically significantly different for the two software solutions (paired *t-*test results shown in the plot, number of compared pairs was *n* = 117 for each case)

Second, as a part of the reproducibility analysis, we wanted to test whether there was a difference in the variability of the results when the analysis was done by different users and compare that to the variability for ImageJ Gels plugin. For that, to quantify the variability, we used the standard deviation of the normalized estimated expression or protein content (data from Fig. 3). An example variability analysis for the image in Fig. 3A is shown in Fig. 4A. As shown in Fig. 4A, the variability depends on the lane with the more difficult lanes showing larger deviation in results obtained by the users. This was the same for the both software solutions. According to our analysis of all the recordings used (Fig. 4B), there are no statistically significant differences in variability between ImageJ Gels and IOCBIO Gel when users are asked to analyze the data for the first time. Thus, in variability of the results obtained by different users, IOCBIO Gel matches the established solution.

### Comparison with available open-source software

In our lab, we started by using the ImageJ Gels plugin [2] for Western blotting analysis. It was relatively easy to use, and it did a good job when we had a few images to analyze. However, as we got more and more experiments to analyze, the rigidity of the plugin and the manual insertion of the values from the signal intensity measurements into our database became too time-consuming. In IOCBIO Gel, the measurements do not have to be re-typed, as the data are exported to either a spreadsheet or, in our case, to our database.

When comparing IOCBIO Gel with ImageJ Gels plugin, there is a major difference in the way how the results are handled. ImageJ Gels plugin analyzes the results in the image-by-image manner. Thus, if one wants to relate the protein(s) of interest measurements to loading controls, the results have to be combined by the user from separate spreadsheets exported by ImageJ. To link that result to some sample ID, these spreadsheets would have to be tagged with the sample IDs as well. In IOCBIO Gel, sample information on the physical gel lane is entered separately and automatically linked to all measurements produced by the program. As a result, following the example above, it is trivial to relate the protein(s) of interest measurements to loading controls by combining data from different images of the same sample probe at the statistical analysis stage.

The other major difference between IOCBIO Gel and other open-source solutions is in the way data are organized. Namely, all measurements related to a scientific study can be grouped together into a project, and it is simple to analyze data either by pulling it directly from the database or by exporting the whole project into a spreadsheet. This functionality is absent in ImageJ and has to be implemented by users through their data processing and organization separately.

IOCBIO Gel offers a fast way to re-analyze a dataset in the case when some correction is needed, such as changes in background subtraction or lane selection. Using the ImageJ Gels plugin, the user has to not only repeat the analysis, but all the new values have to be re-typed into our database or spreadsheets. With IOCBIO Gel, the data are re-analyzed, and the measurement values are updated in the database without the need for re-typing anything. The new data are quickly fetched using the same SQL statement as before. Thus, whereas ImageJ Gels works just fine if you have a few images to analyze, IOCBIO Gel is faster and more foolproof to use with small as well as large datasets. Taking into account that IOCBIO Gel tracks all the steps done while analyzing the gel, it ensures the reproducibility of the analysis at the same time.

Thus, through the automated link between measurements and metadata, IOCBIO Gel leads to a faster workflow that is less prone to human error and allows all data to be stored in the same location. In addition, research laboratories are often joined by people for a short period, by students, post-docs, or even short-term interns. The use of a IOCBIO Gel with its central storage makes it possible to drastically limit the loss of data while offering the possibility of correcting the analysis, even after a person has left the laboratory.

### Availability

In addition to the availability in source code form, the software is distributed as a prepared self-containing archive for MS Windows. For Linux and Mac OS, the software is distributed through the PyPi Python software repository. For installation instructions, see instructions at https://iocbio.gitlab.io/gel.

### Future directions

One of the expected development directions would involve support for other database backends and image sources. The current selection was based on the expertise of our team and its use in our work environment. This includes the use of SQLite and PostgreSQL databases. However, support for the other databases is simple to add if it is supported by SQLAlchemy and if there is an interest. Other image sources can be envisioned as well, and their support will also be demand-driven.

Another line of further development is related to publishing the data. We plan to implement two separate export options for either targeting collaboration or assistance in the journal review process. As the application collects images and metadata describing gels, it should be possible to export the data subset into an archive. Such an archive would be possible to send or distribute online, allowing others to examine and review the gel analysis steps used in the study together with the original gel images. In addition to the archive, we plan to implement report generation for assisting journals with the review of Western Blot and similar images. Many journals request authors to provide labeled Western blot images that would allow the journal to review the analysis steps. We plan to implement the generation of such labeled images that would tag the lanes and the used regions allowing users to provide the labeled images together with the original ones for journals to review.

Other further development will be planned based on the bottom-up approach, driven by the feedback of the users.

## Conclusions

We have developed IOCBIO Gel software that simplifies analysis and metadata entry related to analyzing Western Blot images. The software is not limited to Western blots and can be used to analyze any experimental data leading to a similar representation, i.e., the relationship between the image intensity and the sample. The software allows researchers to enter the data, make all the required image adjustment steps, and select areas of interest. All analysis steps and selections can be adjusted later and reviewed with ease. As a result of the analysis, the overall intensities are given and are associated with the samples in a simple way to incorporate into further statistical analysis.

The developed software addresses major shortcomings in analyzing specific but commonly used experimental protocols and ensures data analysis reproducibility used in these protocols.

## Methods

### Analysis of images

Images representing GAPDH and LTCC expression as well as protein content estimation using Ponceau staining taken from earlier measurements performed for [16]. From the dataset in that study, three membrane images were selected randomly and analyzed by three users. Each user selected the way for analysis as they commonly do, without any specific instructions. For comparison of results between ImageJ Gels plugin and IOCBIO Gel, estimated intensities for a lane were normalized by average intensity of all lanes obtained for that image by user using specific software. Thus, average intensities were found for each image/user/software combination. ImageJ Gels plugin was used as described in [17].

### Statistics

If not stated otherwise, statistics are reported using means ± SD. *p* < 0.05 was considered statistically significant. Differences between estimations performed by IOCBIO Gel and ImageJ Gels plugin were analyzed using paired samples *t*-test. Statistical analysis was performed in R [18].

### Supplementary Information

**Additional file 1.** Gel membrane images used for testing the software. Description of the data: Compressed archive contains images acquired from the same gel membrane either with Ponceau staining or after labeling with antibodies (GAPDH or LTCC). Name of each file in the archive contains the gel numbering and corresponding signal descriptor.

## Data Availability

All data generated or analyzed during this study are included in this published article and its supplementary information files.
