# Supplementary figures and images for: Simple analysis of gel images with IOCBIO Gel
Source: BMC Biol. 2023 Oct 20;21:225. doi: 10.1186/s12915-023-01734-8 (PMC10589977; doi:10.1186/s12915-023-01734-8)

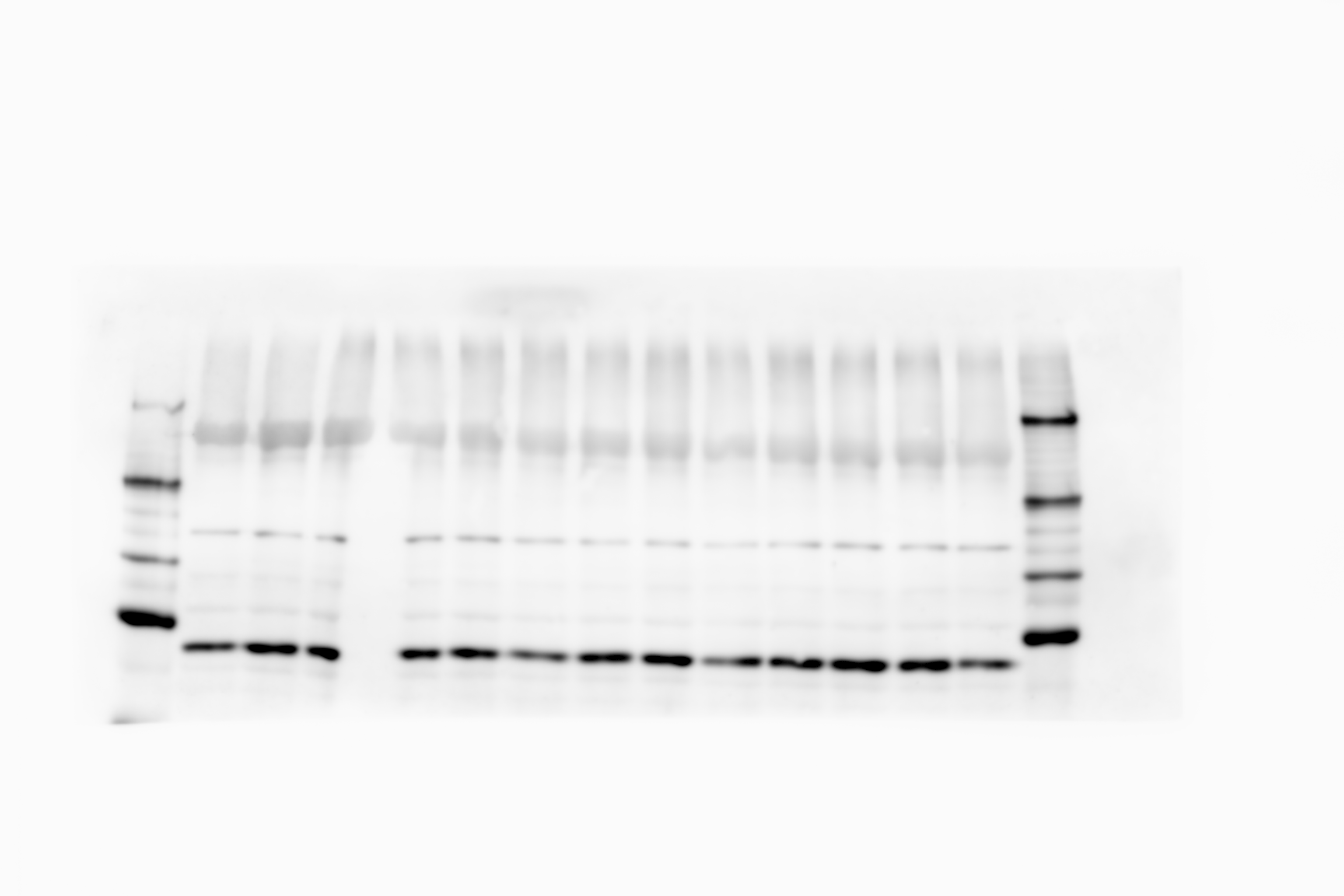

Supplement: Supplementary file 1 — Additional file 1. Gel membrane images used for testing the software. Description of the data: Compressed archive contains images acquired from the same gel membrane either with Ponceau staining or after labeling with antibodies (GAPDH or LTCC). Name of each file in the archive contains the gel numbering and corresponding signal descriptor. [file 12915_2023_1734_MOESM1_ESM.zip › gel_images/gel2_LTCC_expo10min.tif]

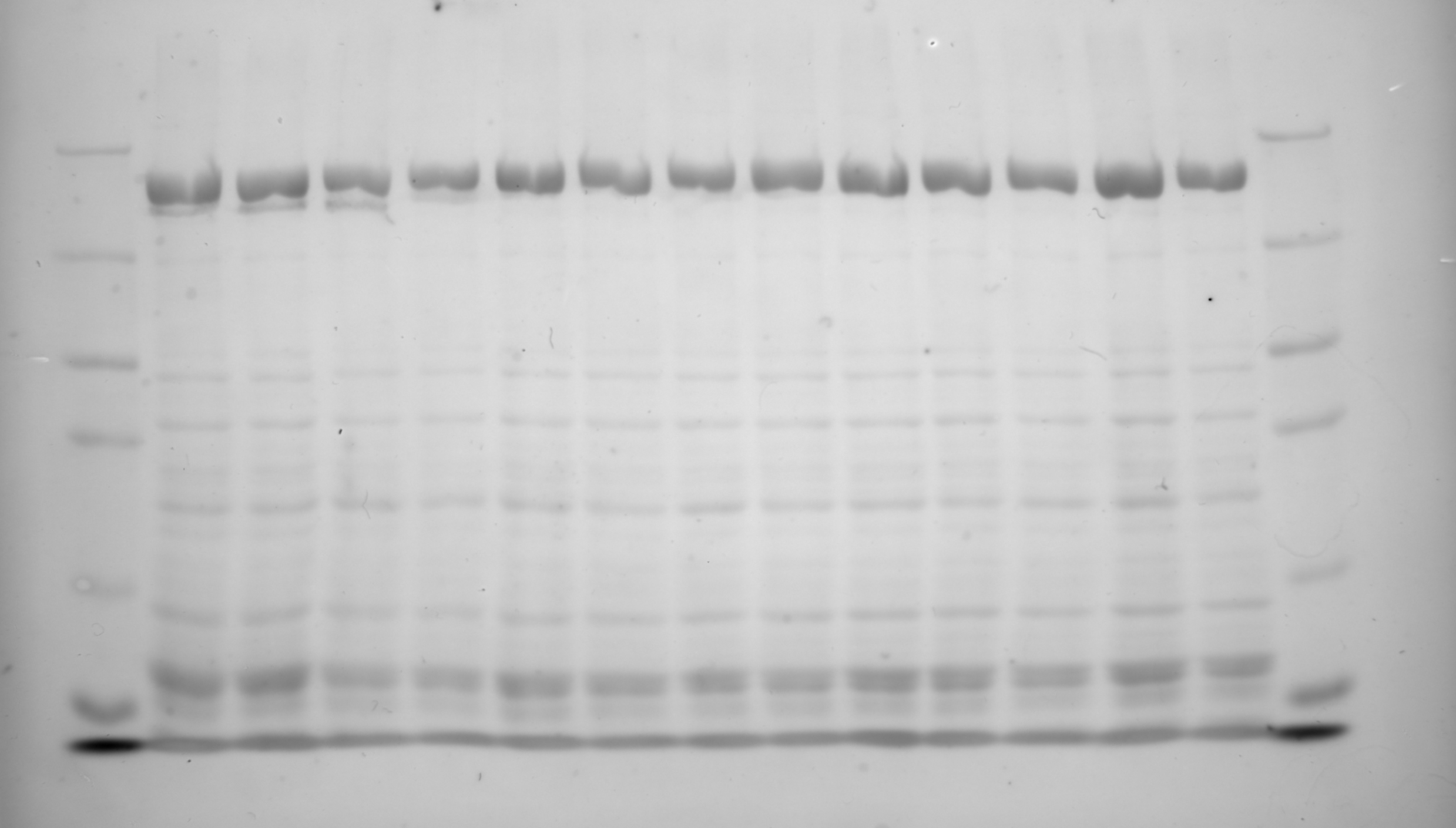

Supplement: Supplementary file 1 — Additional file 1. Gel membrane images used for testing the software. Description of the data: Compressed archive contains images acquired from the same gel membrane either with Ponceau staining or after labeling with antibodies (GAPDH or LTCC). Name of each file in the archive contains the gel numbering and corresponding signal descriptor. [file 12915_2023_1734_MOESM1_ESM.zip › gel_images/gel3_AVG_Ponceau.tif]

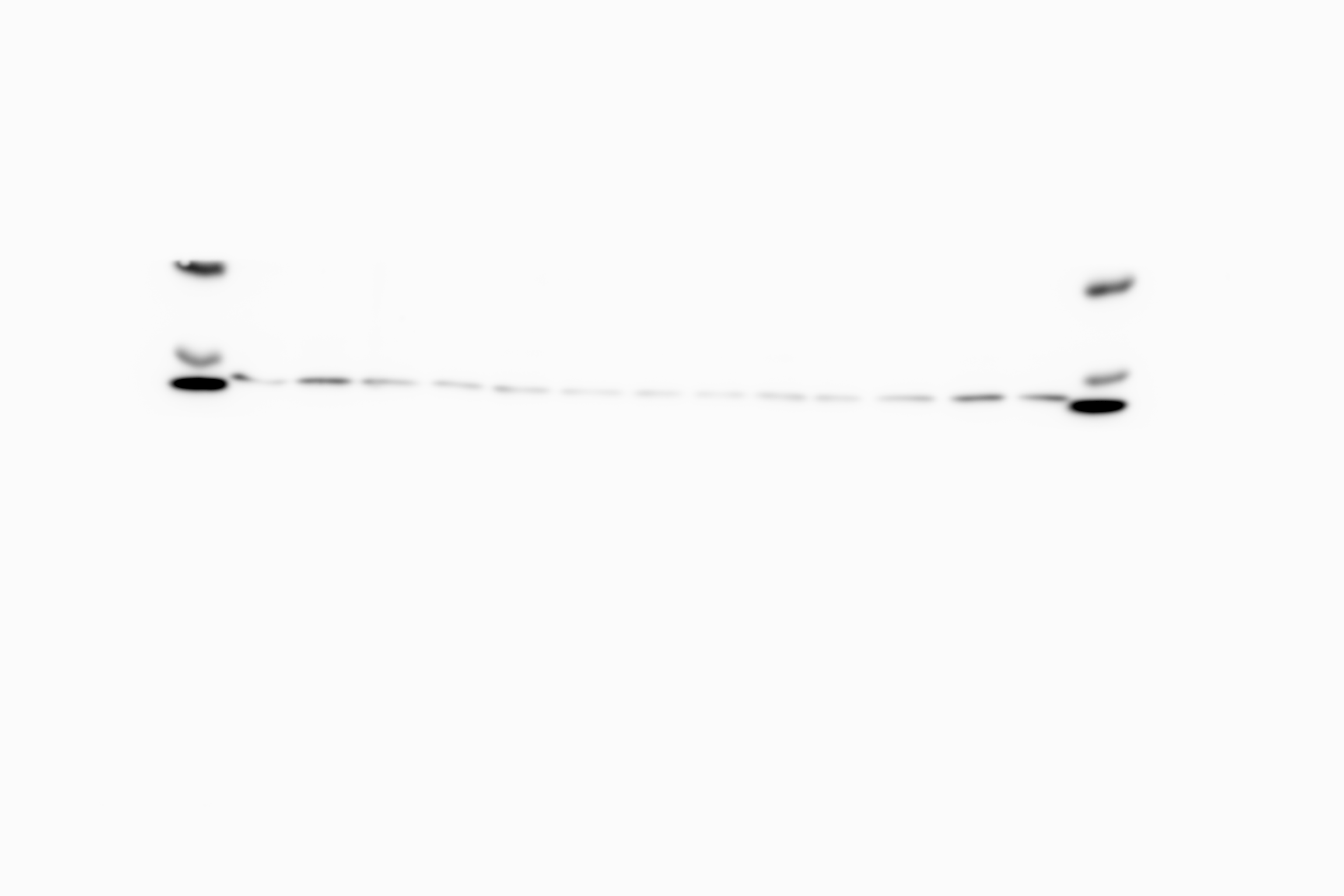

Supplement: Supplementary file 1 — Additional file 1. Gel membrane images used for testing the software. Description of the data: Compressed archive contains images acquired from the same gel membrane either with Ponceau staining or after labeling with antibodies (GAPDH or LTCC). Name of each file in the archive contains the gel numbering and corresponding signal descriptor. [file 12915_2023_1734_MOESM1_ESM.zip › gel_images/gel3_GAPDH_expo3min.tif]

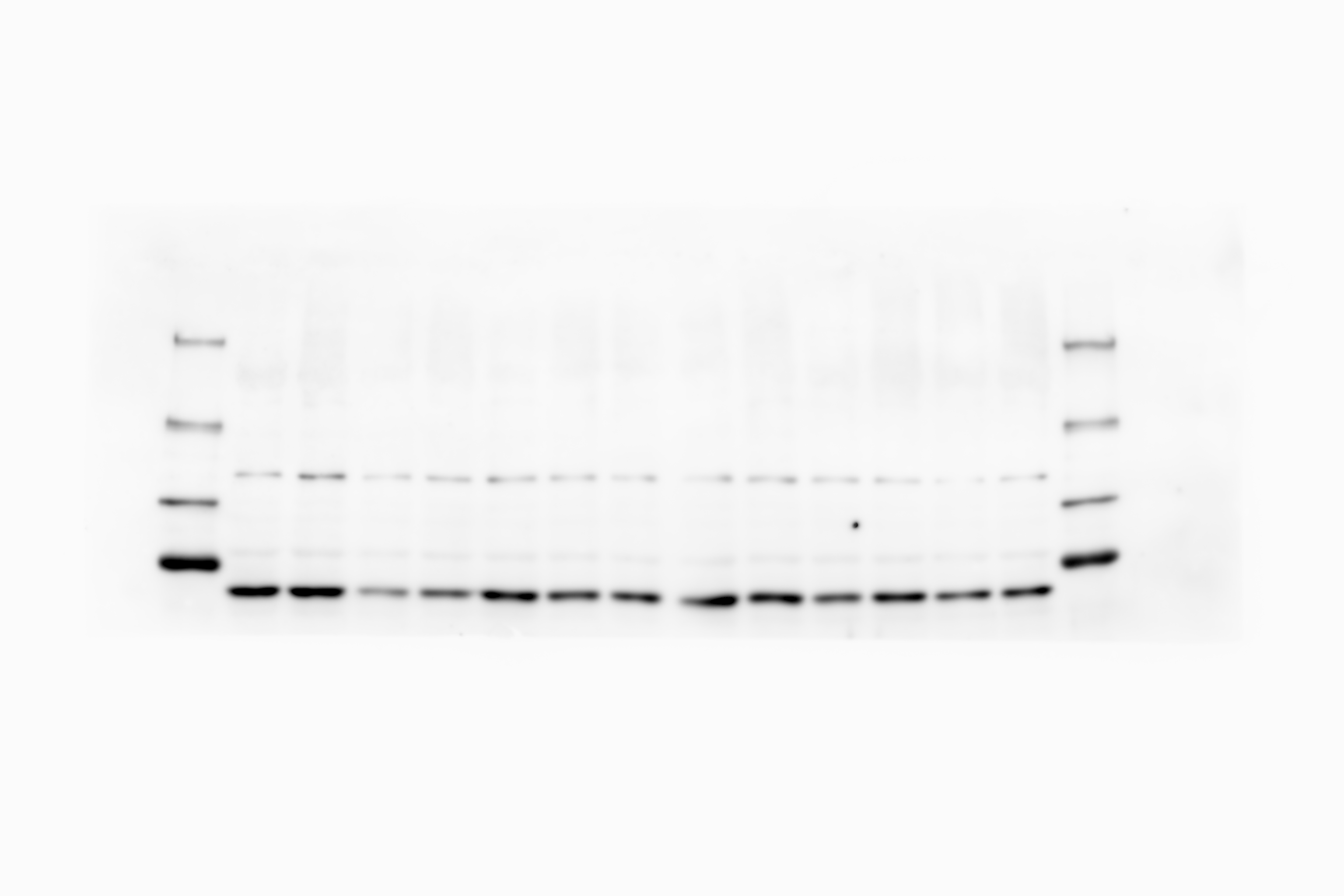

Supplement: Supplementary file 1 — Additional file 1. Gel membrane images used for testing the software. Description of the data: Compressed archive contains images acquired from the same gel membrane either with Ponceau staining or after labeling with antibodies (GAPDH or LTCC). Name of each file in the archive contains the gel numbering and corresponding signal descriptor. [file 12915_2023_1734_MOESM1_ESM.zip › gel_images/gel1_LTCC_expo5min.tif]

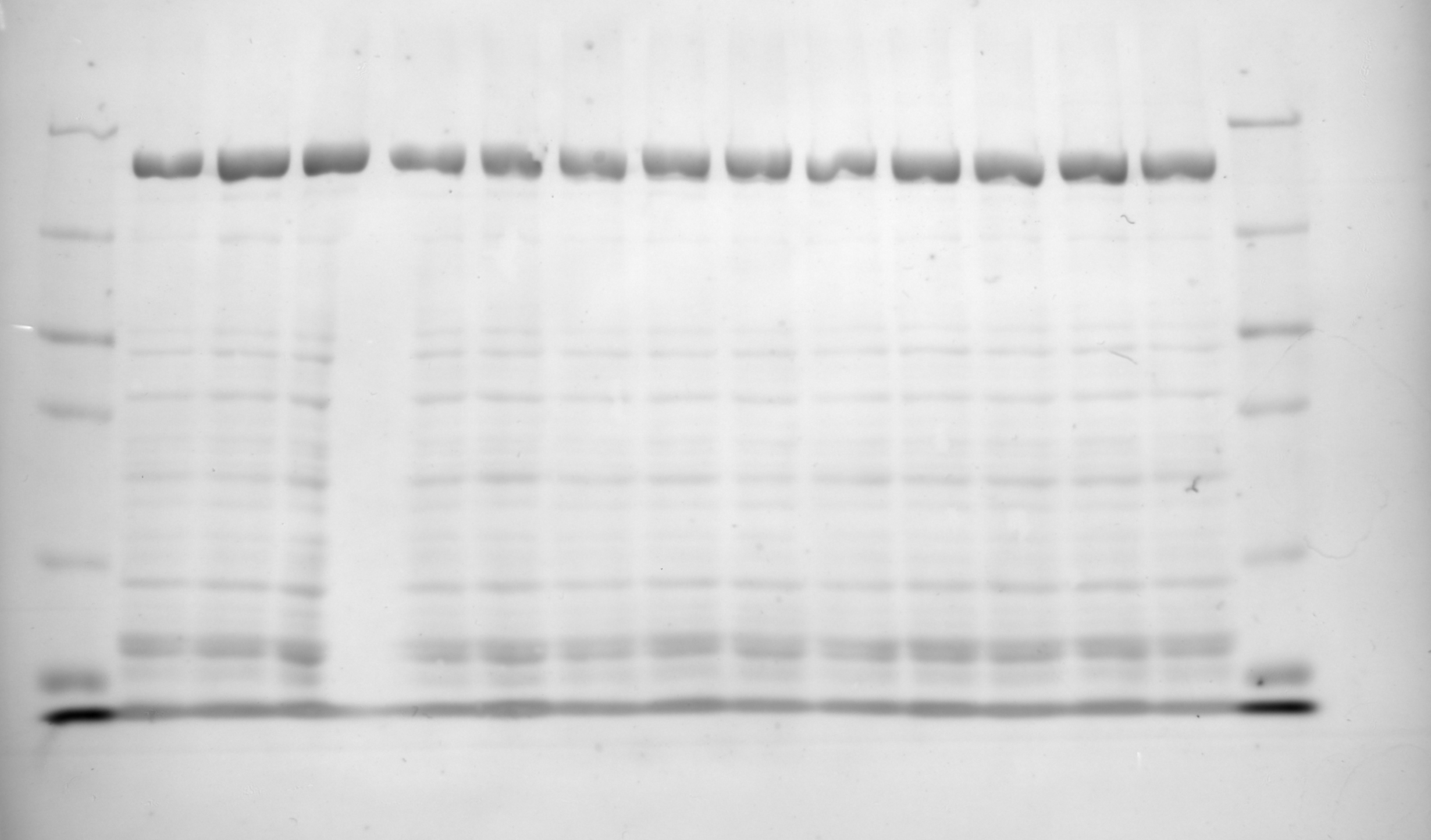

Supplement: Supplementary file 1 — Additional file 1. Gel membrane images used for testing the software. Description of the data: Compressed archive contains images acquired from the same gel membrane either with Ponceau staining or after labeling with antibodies (GAPDH or LTCC). Name of each file in the archive contains the gel numbering and corresponding signal descriptor. [file 12915_2023_1734_MOESM1_ESM.zip › gel_images/gel2_AVG_Ponceau.tif]

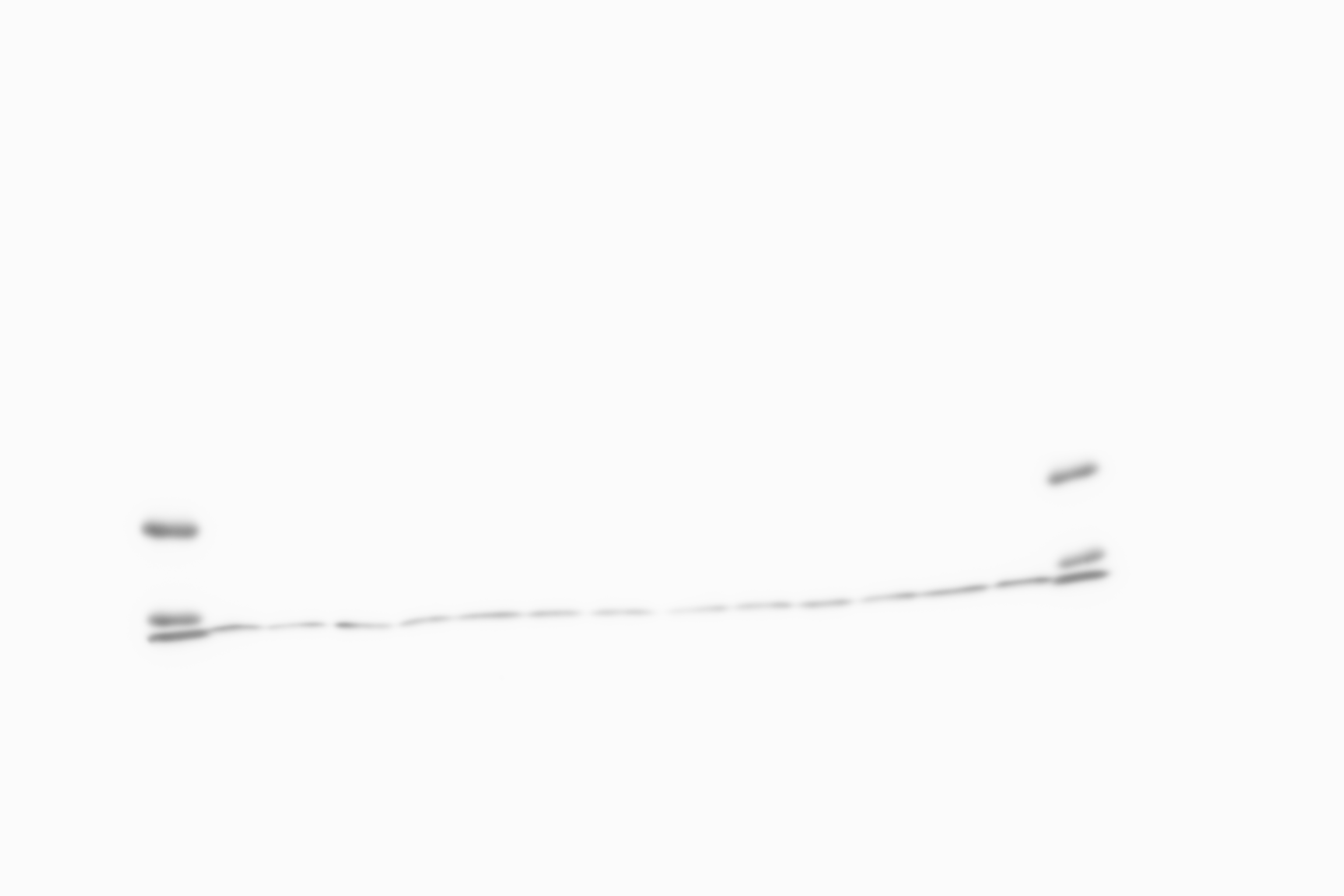

Supplement: Supplementary file 1 — Additional file 1. Gel membrane images used for testing the software. Description of the data: Compressed archive contains images acquired from the same gel membrane either with Ponceau staining or after labeling with antibodies (GAPDH or LTCC). Name of each file in the archive contains the gel numbering and corresponding signal descriptor. [file 12915_2023_1734_MOESM1_ESM.zip › gel_images/gel1_GAPDH_expo1min.tif]

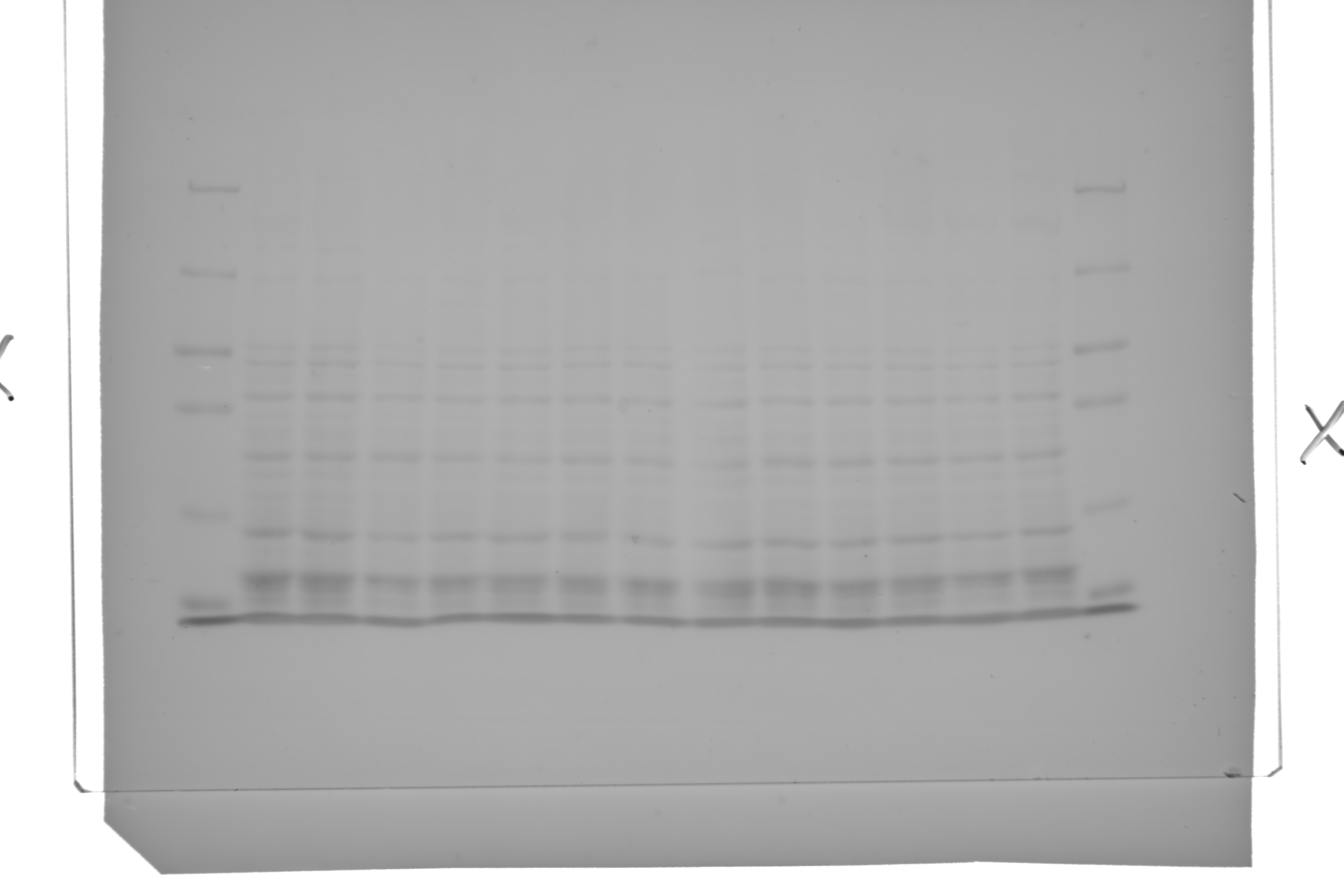

Supplement: Supplementary file 1 — Additional file 1. Gel membrane images used for testing the software. Description of the data: Compressed archive contains images acquired from the same gel membrane either with Ponceau staining or after labeling with antibodies (GAPDH or LTCC). Name of each file in the archive contains the gel numbering and corresponding signal descriptor. [file 12915_2023_1734_MOESM1_ESM.zip › gel_images/gel1_AVG_Ponceau.tif]

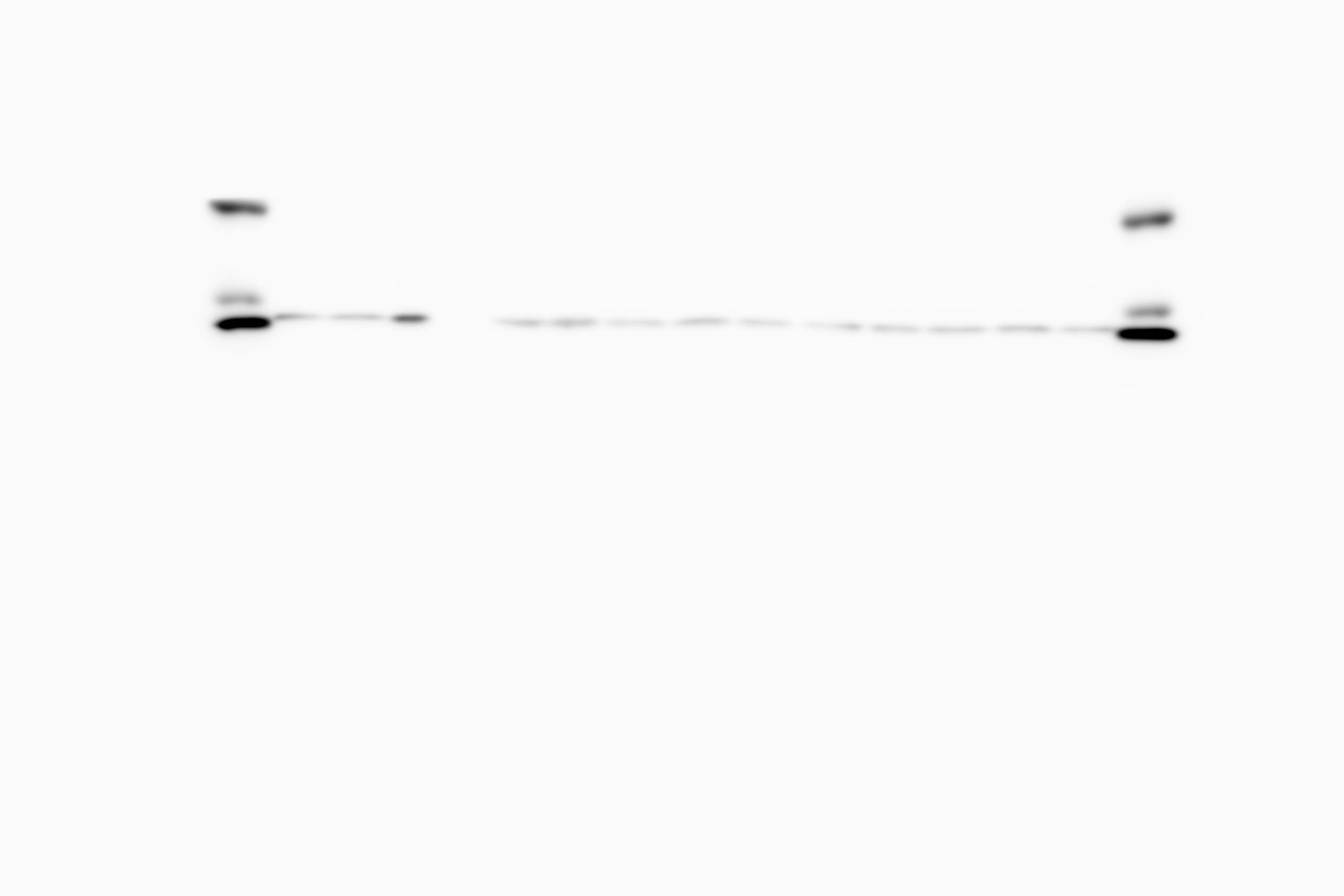

Supplement: Supplementary file 1 — Additional file 1. Gel membrane images used for testing the software. Description of the data: Compressed archive contains images acquired from the same gel membrane either with Ponceau staining or after labeling with antibodies (GAPDH or LTCC). Name of each file in the archive contains the gel numbering and corresponding signal descriptor. [file 12915_2023_1734_MOESM1_ESM.zip › gel_images/gel2_GAPDH_expo3min.tif]

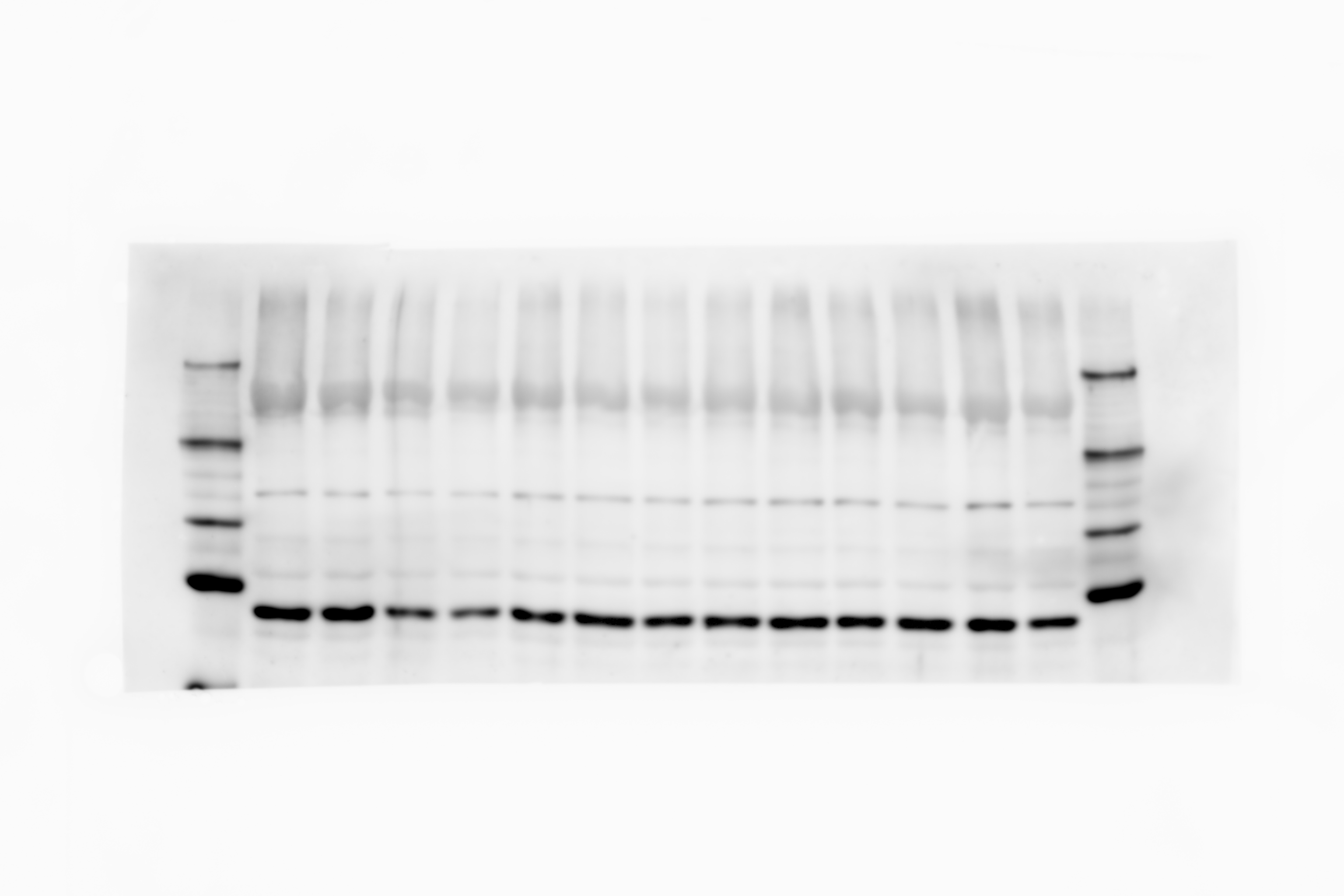

Supplement: Supplementary file 1 — Additional file 1. Gel membrane images used for testing the software. Description of the data: Compressed archive contains images acquired from the same gel membrane either with Ponceau staining or after labeling with antibodies (GAPDH or LTCC). Name of each file in the archive contains the gel numbering and corresponding signal descriptor. [file 12915_2023_1734_MOESM1_ESM.zip › gel_images/gel3_LTCC_expo15min.tif]
